# Comparison of plasma and cerebrospinal fluid proteomes identifies gene products guiding adult neurogenesis and neural differentiation in birds

**DOI:** 10.1038/s41598-021-84274-x

**Published:** 2021-03-05

**Authors:** Eleni Voukali, Nithya Kuttiyarthu Veetil, Pavel Němec, Pavel Stopka, Michal Vinkler

**Affiliations:** Department of Zoology, Faculty of Science, Charles University, Viničná 7, 128 44 Prague, Czech Republic

**Keywords:** Cell biology, Evolution, Zoology, Biomarkers, Neuroscience, Neurogenesis, Proteome informatics

## Abstract

Cerebrospinal fluid (CSF) proteins regulate neurogenesis, brain homeostasis and participate in signalling during neuroinflammation. Even though birds represent valuable models for constitutive adult neurogenesis, current proteomic studies of the avian CSF are limited to chicken embryos. Here we use liquid chromatography–tandem mass spectrometry (nLC-MS/MS) to explore the proteomic composition of CSF and plasma in adult chickens (*Gallus gallus*) and evolutionarily derived parrots: budgerigar (*Melopsittacus undulatus*) and cockatiel (*Nymphicus hollandicus*). Because cockatiel lacks a complete genome information, we compared the cross-species protein identifications using the reference proteomes of three model avian species: chicken, budgerigar and zebra finch (*Taeniopygia guttata*) and found the highest identification rates when mapping against the phylogenetically closest species, the budgerigar. In total, we identified 483, 641 and 458 unique proteins consistently represented in the CSF and plasma of all chicken, budgerigar and cockatiel conspecifics, respectively. Comparative pathways analyses of CSF and blood plasma then indicated clusters of proteins involved in neurogenesis, neural development and neural differentiation overrepresented in CSF in each species. This study provides the first insight into the proteomics of adult avian CSF and plasma and brings novel evidence supporting the adult neurogenesis in birds.

## Introduction

The central nervous system (CNS) function is regulated by a vivid communication with other tissues. This is also achieved through the cerebrospinal fluid (CSF) that supports the brain cell functions^1^. Being from ~ 80% plasma (PL)-derived and ~ 20% brain-cell-derived^2^, CSF dynamically reflects both peripheral and CNS physiological processes. Despite the paramount importance of the relationship between blood and CSF, little comparative effort has been made in non-mammalian vertebrates to characterise the two vital fluids integrating processes in and out of the CNS.

There is widespread evidence that the birth and growth of neurons also occurs in adult invertebrate and vertebrate CNS and that these cells add onto existing neuronal networks or replace apoptotic neurons^3^. In addition, several in vitro and in vivo studies have provided evidence that, CSF can promote neurogenesis even in the adult brain^4–9^. In particular, spatial gradients of CSF proteins induced the migration of neuroblasts from the subventricular zone to the olfactory bulb in the adult mouse brain^8^. Also, when rat CSF was applied as a culture medium to neural stem cells and cortical explants, the cells survived without additional supplements, particularly for age-matched CSF samples and tissues^9^. Therefore, increased interest has been raised to study and describe the molecular composition of CSF, especially in humans^10–16^.

Although CSF proteomic composition shares many similarities across species such as rat, human and chicken^6, 10–12^, species differ in physiological requirements of their tissues and different species show different neurogenesis rates as well as anatomical features^11, 17^. Birds have been proposed as suitable models to investigate the molecular basis of the regulation of adult constitutive neurogenesis^18^. Nonetheless, the available avian CSF proteomic analyses have addressed only embryonic chicken CSF. Based on two-dimensional polyacrylamide gel electrophoresis (2D-PAGE) followed by mass spectrometry (MS) or tandem mass spectrometry (MS/MS) methods, the chicken embryonic CSF (eCSF) consists of hundreds of proteins of the extracellular matrix, regulators of osmotic pressure, ion carriers, hormone-binding proteins, regulators of lipid metabolism, and various enzymes and their regulators^6, 10, 11^. However, the advent of methods such as gel-free-LC/MS for comparative protein analysis has resulted in several advantages over the label-based methods used by the aforementioned studies, including the greater coverage of the proteome^19^.

While the domestic chicken (*Gallus gallus*, GG, order Galliformes) serves as a basic avian model organism in developmental biology and neuroscience^20, 21^, current evidence shows that its neural densities are much lower than in some other, evolutionarily derived birds^22, 23^. Importantly, recently, parrots (order Psittaciformes) have been proposed as models for neurobiological research^24^. Similarly to humans, parrots have large brains relative to their body size^25^, high density of neurons in the forebrain^22^, exhibit outstanding cognitive skills such as object permanence^26, 27^ and tool use^26, 28^, cooperative problem solving^29^, complex social organization^30^, learning of vocalizations through cultural transmission^31, 32^, and some species are on par with great apes in number of cognitive domains^33–35^. Furthermore, despite their high metabolism, they show extended developmental periods^36^ and longevity^24, 37^.

In the present study, we focus for the first time on avian adult CSF and PL proteomes, comparing the domestic chicken to two parrot species: the budgerigar (*Melopsittacus undulatus*, MU) and the cockatiel (*Nymphicus hollandicus*, NH), representing two distinct parrot clades (Psittacoidea and Cacatuoidea, respectively^38^). Since the cockatiel genome is not presently available, a condition that may affect the peptide/protein identification, we first test the consistency of the proteomic annotation by comparing the MS/MS identification success rates after mapping all spectra against the reference proteomes of three model avian species: chicken, budgerigar and zebra finch (*Taeniopygia guttata*). Second, using the quality-filtered and consistent data, we describe the proteomic profiles of PL and CSF in the three bird species, searching for the pathways common to differentiate the avian PL and CSF proteomes.

## Results

### Reference selection for proteome mapping

**Figure 1:**
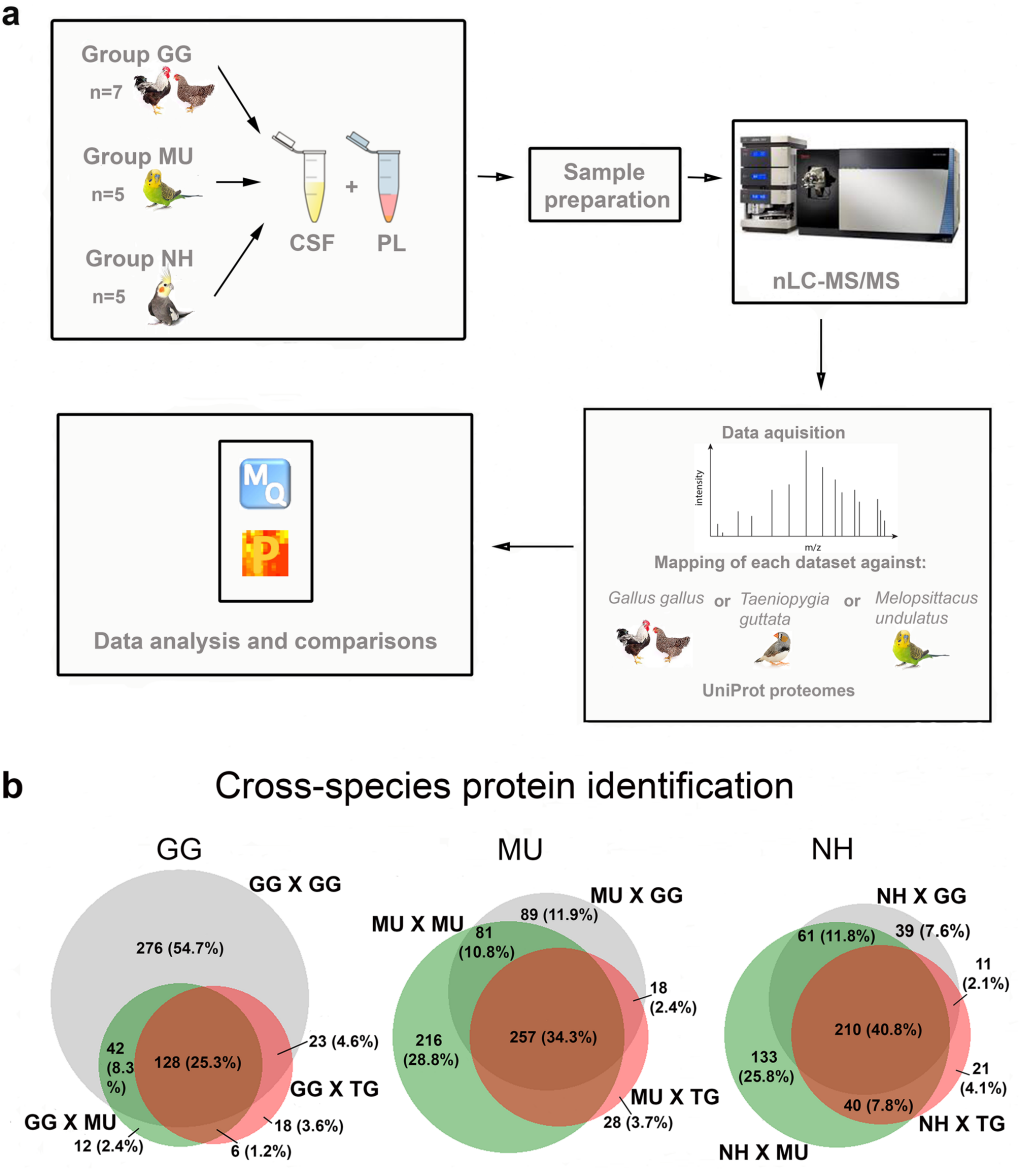
Comparison of the nLC-MS/MS cross-species protein identification success in both cerebrospinal fluid (CSF) and plasma (PL) samples of chickens (GG), budgerigars (MU) and cockatiels (NH), after mapping to three selected avian reference proteomes GG, MU and *Taeniopygia guttata* (TG). (**a**) The overview of the study design and mapping approach is schematically illustrated. (**b**) Venn diagrams show the proportions of proteins identified based on the different reference proteomes of GG (grey), TG (red) and MU (green) for GG, MU and NH (BioVenn, http://www.biovenn.nl/).

We mapped each chicken, budgerigar and cockatiel CSF and PL sample spectra against the chicken, zebra finch and budgerigar reference proteomes (Fig. 1a). The total numbers of spectra, numbers of unique spectra and numbers of the identified peptide sequences obtained for each mapping combination are shown in Supplementary Table S1, along with the total numbers of unique proteins identified for each data set before and after filtering. The list of proteins represented in individual samples together with their abundances and the ortholog gene names corresponding to each identified protein ID is also provided in Supplementary Table S1. We found that a large proportion (25.1%, 35% and 41.1% for chicken, budgerigar and cockatiel, respectively) of orthologous gene IDs overlapped when mapped against chicken, zebra finch and budgerigar reference proteomes in either CSF or PL, being consistent for all avian species examined in this study (Fig. 1b). However, part of the proteins remained unidentified when the CSF and PL spectra were mapped against either the same species proteome or the budgerigar proteome in the case of cockatiel. Regarding chicken spectra, about 7.2% (37) out of the total proteins identified for chicken were not identified when mapped against the chicken reference proteome but found when mapped against zebra finch and budgerigar references; for the budgerigar, 18% (125) of identities were missed when mapped against budgerigar genome, but detected when mapped against the zebra finch or chicken; and for the cockatiel, 13.8% (70) of identities were missed when mapped against the budgerigar proteome, but detected when mapped against the zebra finch and/or chicken. The list of these genes is included in Supplementary Table S1 and contains, among others, members of multigene families such as histones, and myoglobins. A Gene Ontology (GO) classification of the biological functions for each subset of these missing proteins is shown in Fig. S1 in Supplement. The most represented biological functions of these missing proteins were: (1) Cellular processes (61.1, 47.7, 53.3% of the missing proteins in GG, MU and NH, respectively) including the cell communication, cellular component organization, developmental process, homeostasis, metabolic process, response to stimulus, export from cell, movement of cell or subcellular component, protein folding and signal transduction; (2) Biological regulation of molecular functions (27.8, 19.3, 28.9% of the missing proteins in GG, MU and NH, respectively); and (3) Metabolic processes (27.8, 6.4, 35.6% of the missing proteins in GG, MU and NH respectively) including ATP metabolic process, biosynthetic process, catabolic process, cellular metabolic process, nitrogen compound metabolic process, organic substance metabolic process, oxidation–reduction process, primary metabolic process and small molecule metabolic process. Since mappings from multiple annotations could not be combined and a higher proportion of gene products were exclusively identified when the proteomes were mapped against the same species reference proteome (chicken and budgerigar) or a closely related one (cockatiel), we selected only these datasets for further analysis and focused mainly on consistently represented proteins.

### Blood PL and CSF proteomes description

**Figure 2:**
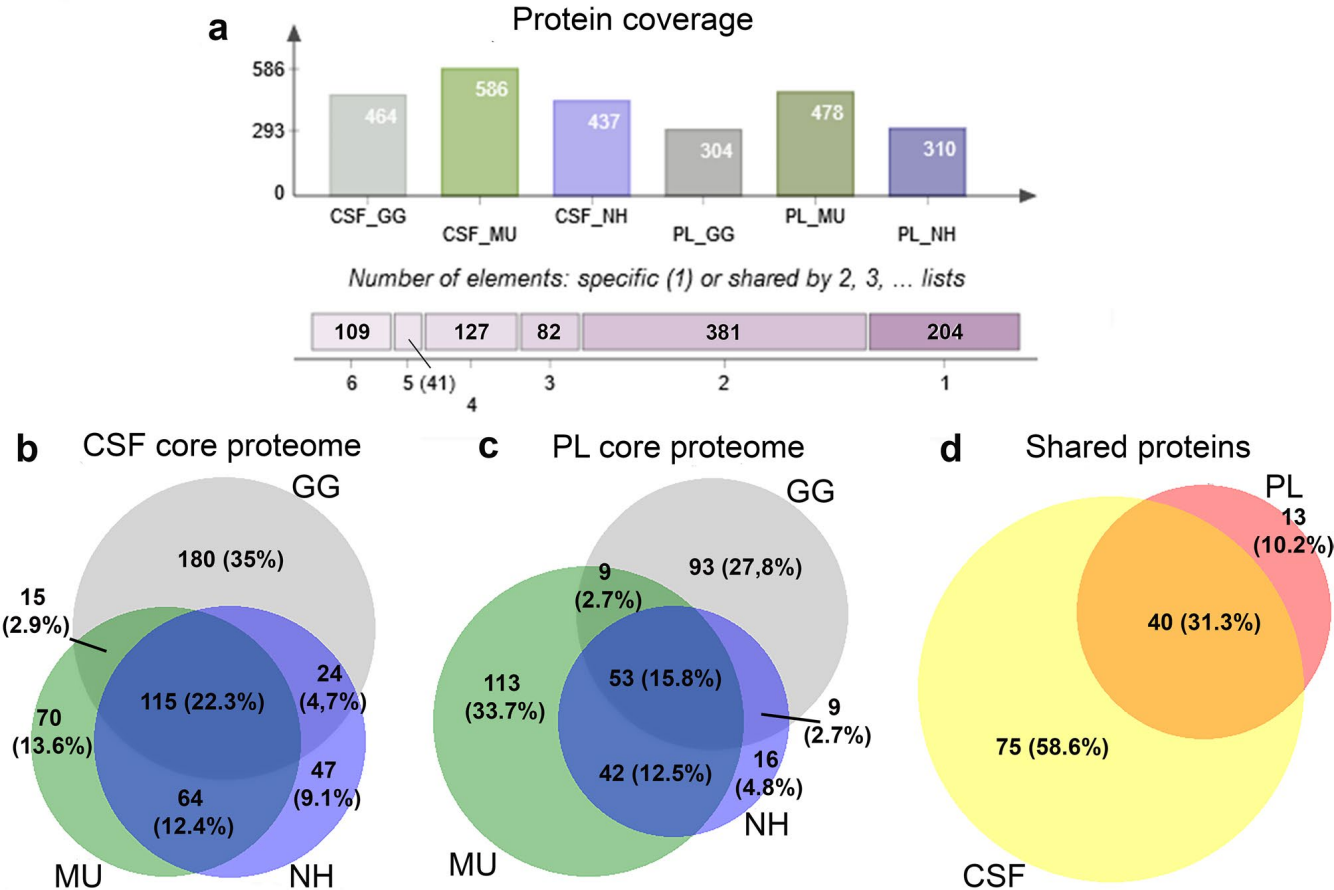
Overview of the proteomic coverage across species and body fluids. The identified plasma (PL) and cerebrospinal fluid (CSF) proteins of chicken (GG), budgerigar (MU) and cockatiel (NH) and the shared identifications between the groups are demonstrated in (**a**) (Venn Diagrams, http://bioinformatics.psb.ugent.be/webtools/Venn/). The Venn diagrams are showing the overlapping numbers and percentages of core proteomes of CSF (**b**) and PL (**c**) for GG, MU and NH, and (**d**) the numbers and percentages of shared proteins across all the three species for CSF and PL (BioVenn, http://www.biovenn.nl/).

In total, after filtering, we identified 464 proteins represented in the chicken CSF proteome, 586 proteins in the budgerigar CSF proteome and 437 proteins in the cockatiel CSF proteome. The PL proteome consisted of 304 proteins in the chicken, 478 in the budgerigar and 310 in the cockatiel. From these, 109 proteins (11.3%) were common to all species (GG, MU, NH) and all sample types (PL or CSF) (Fig. 2a), while a large number of proteins varied even between individual samples of the same tissue and species (Supplementary Fig. S2). The detailed protein coverage of each dataset is shown on Supplementary Fig. S3. We then defined the core proteomes, that is, proteins represented in all conspecific individuals for each fluid. The complete list of the proteins and abundances of the CSF and PL core proteomes for the three species is tabulated in the Supplementary Table S2. Considering the list of proteins detected across all individuals across all species in the particular sample types, we identified 115 common proteins in the CSF (22.3%, Fig. 2b), 53 common proteins in the PL (15.8%, Fig. 2c), and 40 proteins overlapping between CSF and PL (Fig. 2d). The CSF core proteome consisted of 344 proteins (71.22% of the total proteins detected across all individuals) in chicken, 269 proteins (41.97%) in budgerigar and 274 (56.92%) proteins in cockatiel. The PL core proteome consisted of 173 proteins (55.27%) in chicken, 227 proteins (45.31%) in budgerigar and 124 proteins (38.87%) in cockatiel.

**Figure 3:**
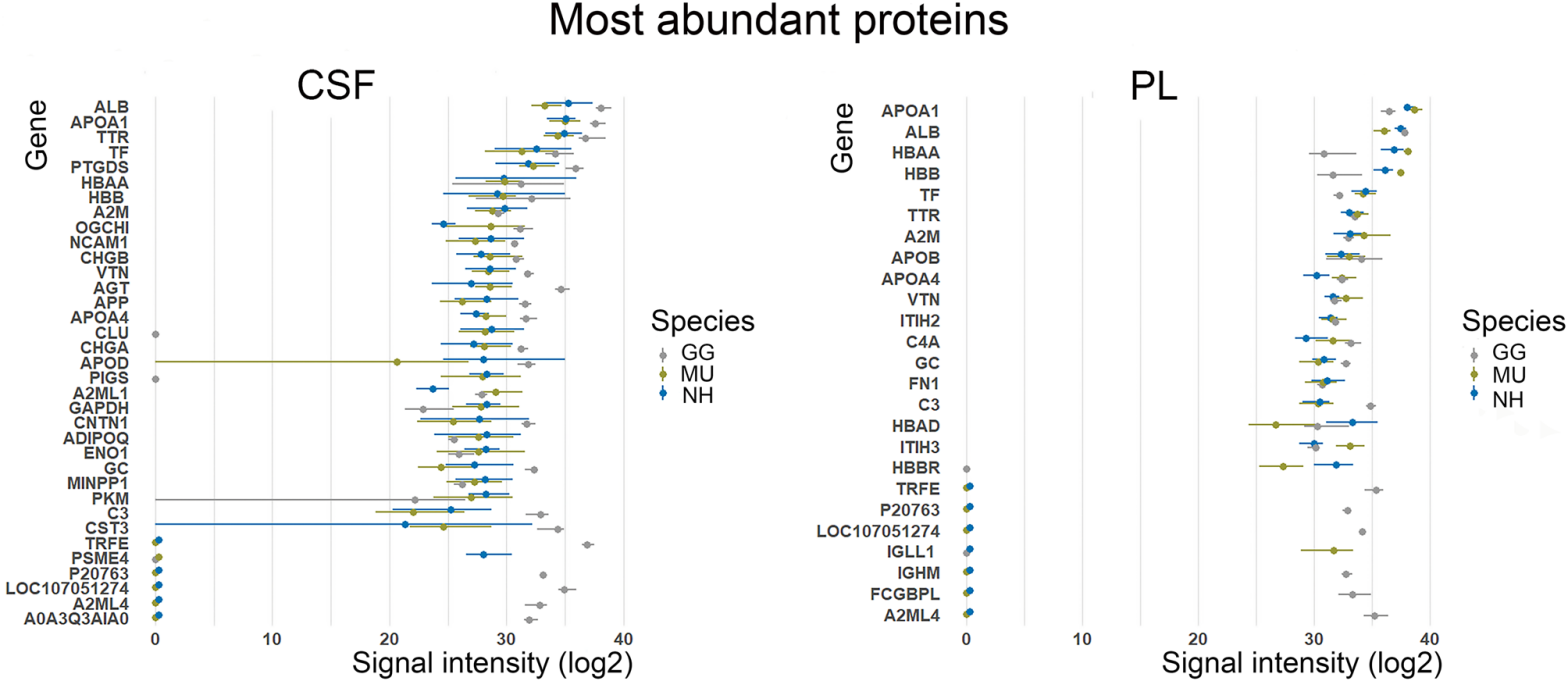
The top 2% most abundant cerebrospinal fluid (CSF) and plasma (PL) proteins of chicken (GG), budgerigar (MU) and cockatiel (NH) are presented using their gene code: GG—grey, MU—green and NH—blue. The logarithmic normalised abundances are shown as a range (minimum–maximum) across all individuals (GG n = 7, MU n = 5, NH n = 5) with a mean highlighted as a dot (R version 4.0.0, www.r-project.org).

We ranked the shared identified proteins according to their normalized logarithmic abundance means. There was a considerable inter-individual variability in the expression of the lowly abundant proteins in both PL and CSF. In contrast, among the highly abundant proteins, the protein composition appears relatively consistent in both fluids between the three species, parrot species being more similar (Fig. 3). Among the 2% most abundant proteins identified in both the fluids and across all the species belongs namely albumin (ALB), ovotransferrin (TF), alpha 2 macroglobulin (A2M), transthyretin (TTR), vitronectin (VTN) and apolipoproteins (APOA1, APOA4).

**Figure 4:**
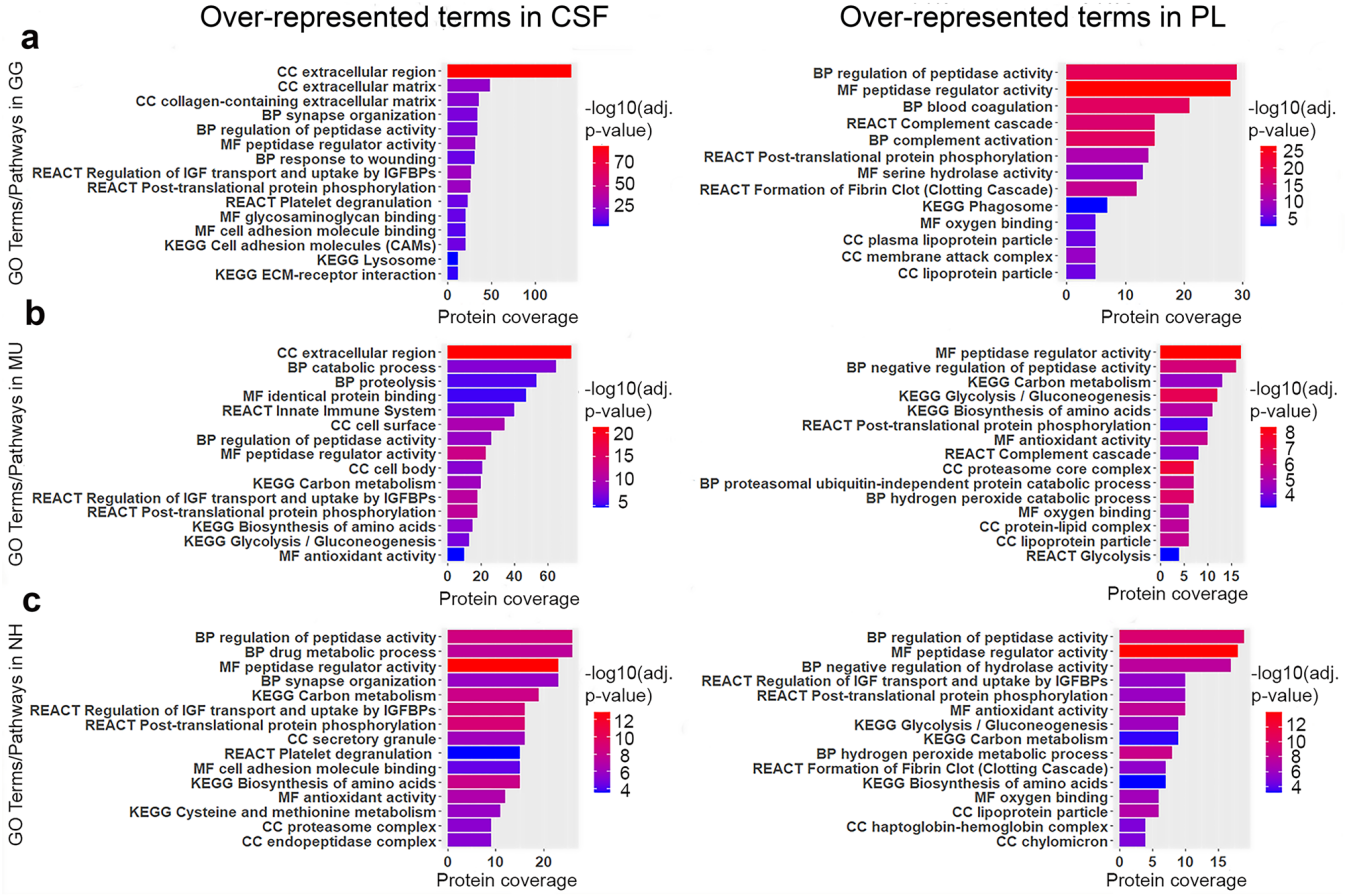
Top significant over-represented pathways revealed after functional enrichment analysis of the cerebrospinal fluid (CSF) and blood plasma (PL). Enriched pathways in CSF are shown on the left-hand side and in PL on the right-hand side for (**a**) chicken (GG), (**b**) budgerigar (MU) and (**c**) cockatiel (NH). The x-axis indicates the numbers of genes represented in each pathway, the y-axis indicates the GO (Molecular function, MF; Biological Process, BP; Cellular component, CC) or Pathways terms (KEGG, REACT) involved, and the colours represent the size of the negative log10 of the adjusted *p* value (adjusted *p* < 0.05). *IGF* Insulin-like Growth Factor; *IGFBPs* Insulin-like Growth Factor Binding Proteins (g:Profiler version e102_eg49_p15_b486be1, https://biit.cs.ut.ee/gprofiler/gost; R version 4.0.0, www.r-project.org).

In general, the proteins commonly represented in CSF and blood PL were involved in metabolic and signalling pathways and most of their functions are common to CSF and plasma (Fig. 4). As expected, CSF proteins are mostly extracellular components expressed into the extracellular space. The complete results of the pathway enrichment analysis are shown in Supplementary Table S3.

### Comparison of the plasma and CSF proteomes

**Figure 5:**
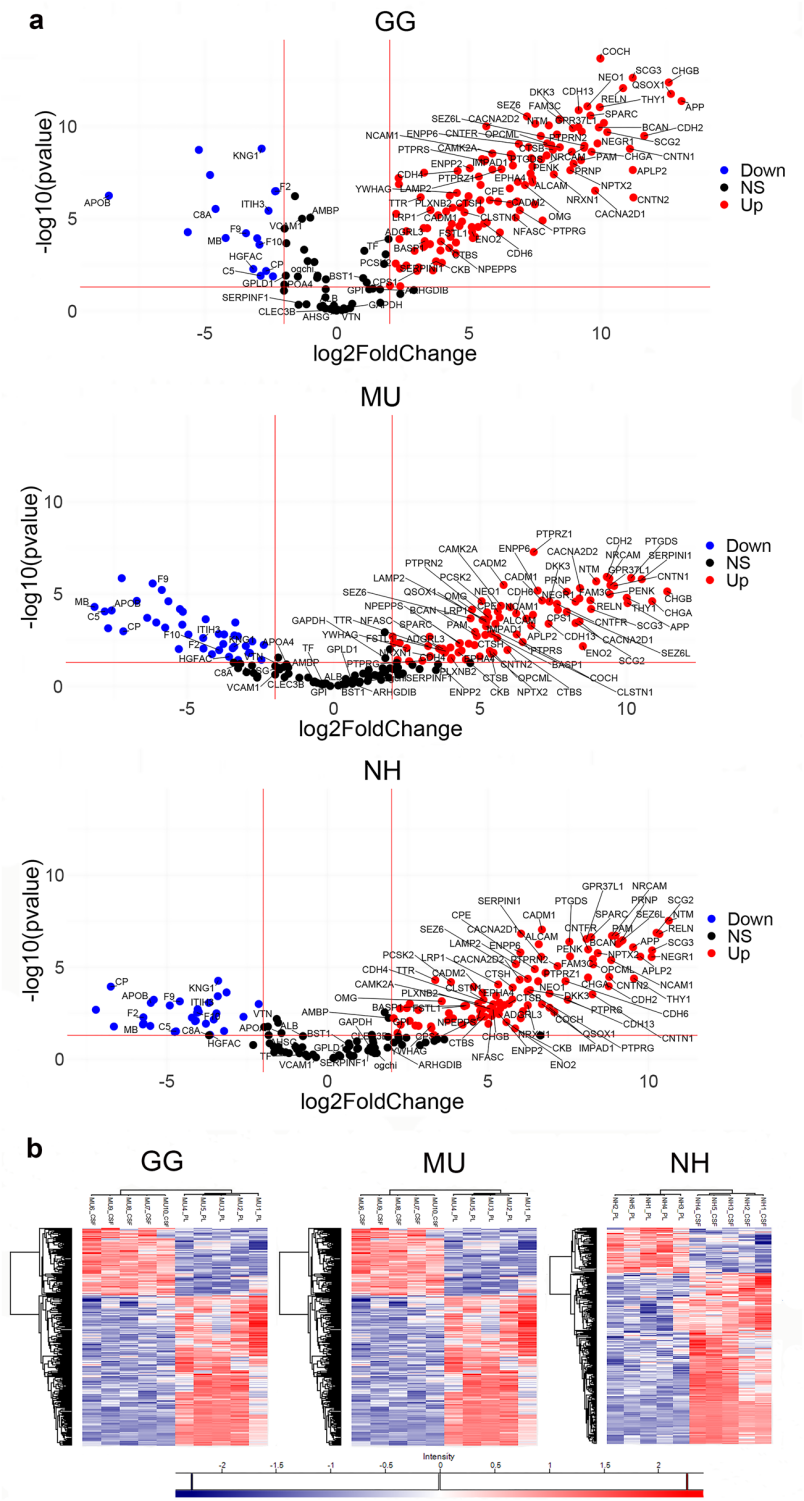
Comparative analyses of cerebrospinal fluid (CSF) and plasma (PL) proteomes. The distribution of the shared proteins across species and clustering of over-represented and down-represented proteins between the two fluids is shown in for chicken (GG), budgerigar (MU) and cockatiel (NH). (**a**) The volcano plots graphically demonstrate the fold changes on *p* values between CSF and PL proteomes that are shared across species, following Student t-tests corrected by the Permutation Based false-discovery rate (FDR) (FDR-adjusted *p* value < 0.05). The fold differences of the proteins (x-axis) dependent on their *p* values (y-axis) are shown in dots, colour-coded in blue, red and black, demonstrating the significantly under-represented proteins (Down), over-represented proteins (Up), and non-significantly represented proteins (NS, *p* < 0.05, fold change cut-off ≥ 2) respectively in CSF compared to PL for GG, MU and NH. The proteins that are commonly differentiated are indicated and labelled using their gene codes (R version 4.0.0, www.r-project.org). (**b**) Hierarchical clustering heatmaps show differences in protein expression between CSF and PL in GG, MU and NH, respectively (*p* < 0.05, after multiple testing). Proteins with high (red) and low (blue) expression form two clusters differentiating the fluids (Perseus version 1.6.10.50, http://www.perseus-framework.org).

Raw and normalised data were inspected by their log2 distributions and correlation of their principal component 1 of the shared genes. After data normalization, the distributions showed almost no variation between the samples and their first principal component positively correlated with the first principal component of raw data (Supplementary Fig. S4). Principal component analyses (PCA) of CSF and PL protein abundances showed that the two fluids form separate clusters for all three studied species (Supplementary Fig. S5). For chicken, the first two principal components related to fluid type explained 84.76% of the total variance; for budgerigar 70.22% and cockatiel 74.21%, indicating that CSF and PL proteomes are distinguishable in these species. The exploration of the differentially represented proteins in CSF and PL proteomes in GG identified 413 significantly differentially abundant proteins, including 49 under-represented proteins and 289 over-represented proteins in CSF compared to PL with false discovery rate (FDR) adjusted *p* value < 0.05 and fold change cut-off ≥ 2. Similarly, 332 proteins were significantly differentially represented in MU CSF compared to PL proteome, including 99 under-represented and 224 over-represented proteins (FDR adjusted *p* value < 0.05, fold change cut-off ≥ 2) and, for NH, the analysis revealed 289 differentially represented proteins, 46 under-represented and 206 over-represented in CSF compared to PL (FDR adjusted *p* value, fold change cut-off ≥ 2). The fold change differences in the common proteins across species are shown in volcano plots at Fig. 5a. The fold change differences in all differentially represented proteins for each species are shown in the Supplementary Fig. S6. Detailed information on these differentially represented proteins is provided in the Supplementary Table S4. The differentiation between protein content of CSF and PL was further indicated based on hierarchical clustering that we used to visualise the groups of differentially represented proteins in either CSF or PL in all the three studied avian species (Fig. 5b).

**Figure 6:**
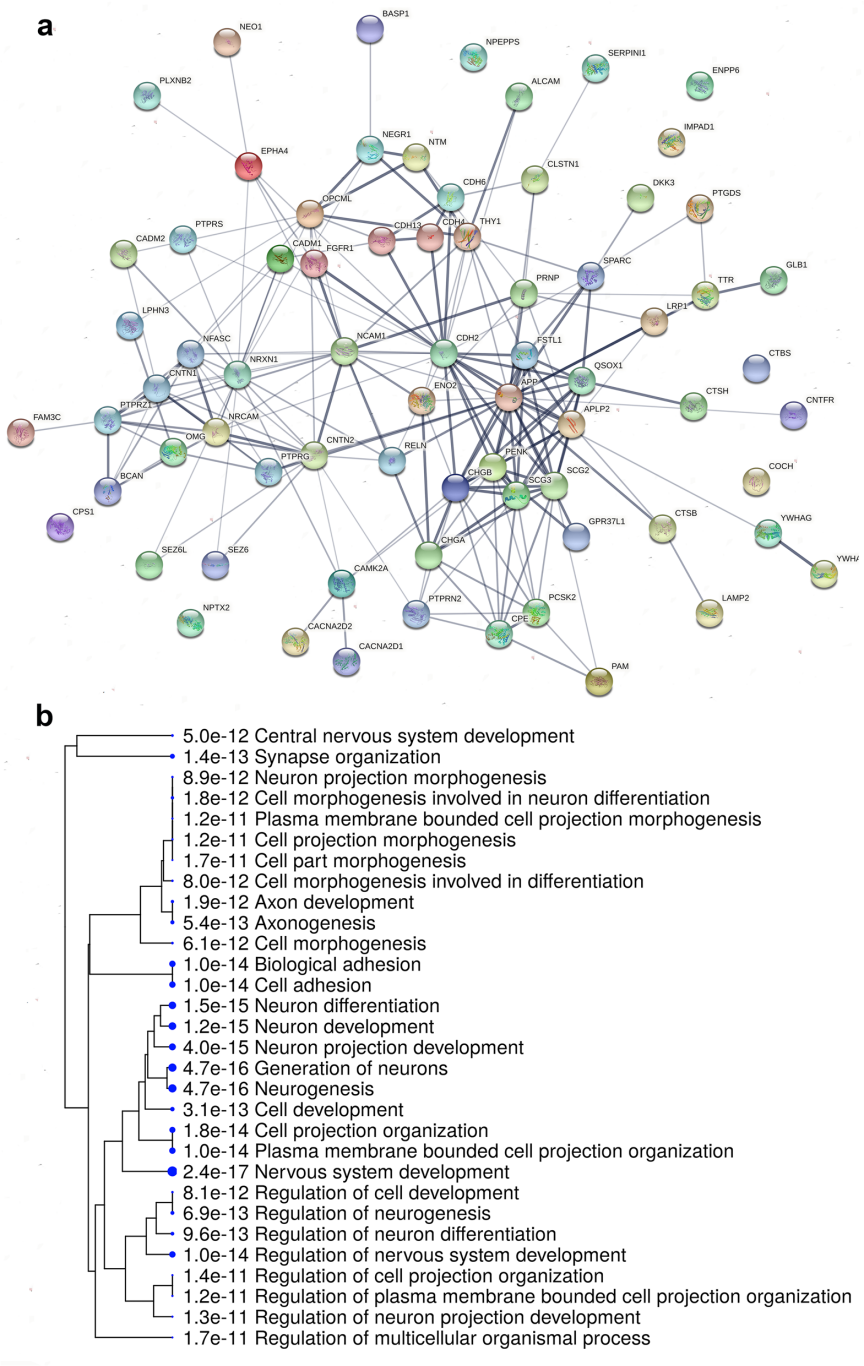
Protein–protein interactions (PPIs) and enriched pathways of 72 over-represented proteins in CSF compared to PL commonly in chicken, budgerigar and cockatiel. (**a**) STRING analysis of the protein interaction network showing associations of the proteins. Line thickness indicates the strength of data support (STRING version 11.0, http://string-db.org). (**b**) Hierarchical clustering tree summarizing the correlation among the significant pathways enriched in the 72 commonly over-represented CSF proteins across the three species. GO terms of biological functions revealed pathways related to neurogenesis and neuron differentiation. Pathways with many shared genes are clustered together and dot sizes indicate the degree of statistical significance (ShinyGO version 0.61, http://bioinformatics.sdstate.edu/go/).

We found that 72 proteins over-represented in CSF compared to PL overlap between GG, MU or NH, belonging to various functional modules such as cell-adhesion proteins, growth factors, subunits of calcium channels etc. Interestingly, four commonly shared CSF proteins (*LCAT*, phosphatidylcholine-sterol acyltransferase; *MCFD2*, multiple coagulation factor deficiency protein 2; *PRDX1*, peroxiredoxin-1) are under-represented in the CSF of parrots but over-represented in chicken. We did not identify any proteins over-represented in the CSF of chicken but under-represented in parrots. STRING analysis of the 72 commonly over-represented proteins in CSF of all species indicates a network of protein interactions, containing 72 nodes and 199 edges (Fig. 6a). The GO Biological function terms related to this protein–protein interaction (PPI) network indicate developmental and neogenesis processes. The same gene set was visualised as a hierarchical clustering tree using ShinyGO (Fig. 6b).

**Figure 7:**
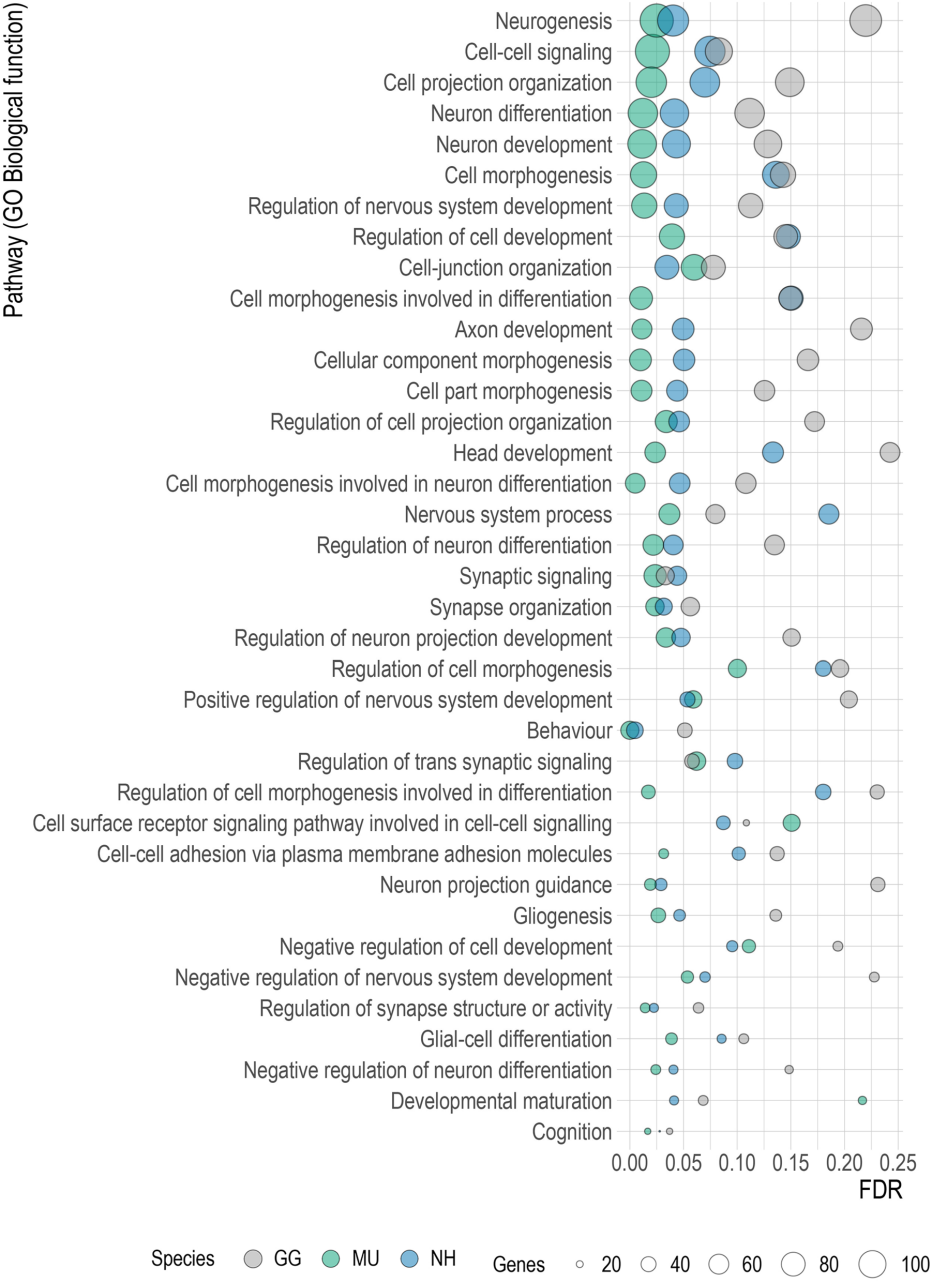
Significant gene sets related to neural function commonly enriched in all species. The dot plot shows the identified enriched terms by GSEA with nominal *p* value < 0.05. *P* values were adjusted for multiplicity of testing. The x-axis shows the range of the false discovery rate (FDR) values, the y-axis lists the GO Biological function terms, the colours indicate the involved species and the dot size shows the number of genes in each set (GSEA version 4.1.0, http://www.gsea-msigdb.org; R version 4.0.0, www.r-project.org).

**Figure 8:**
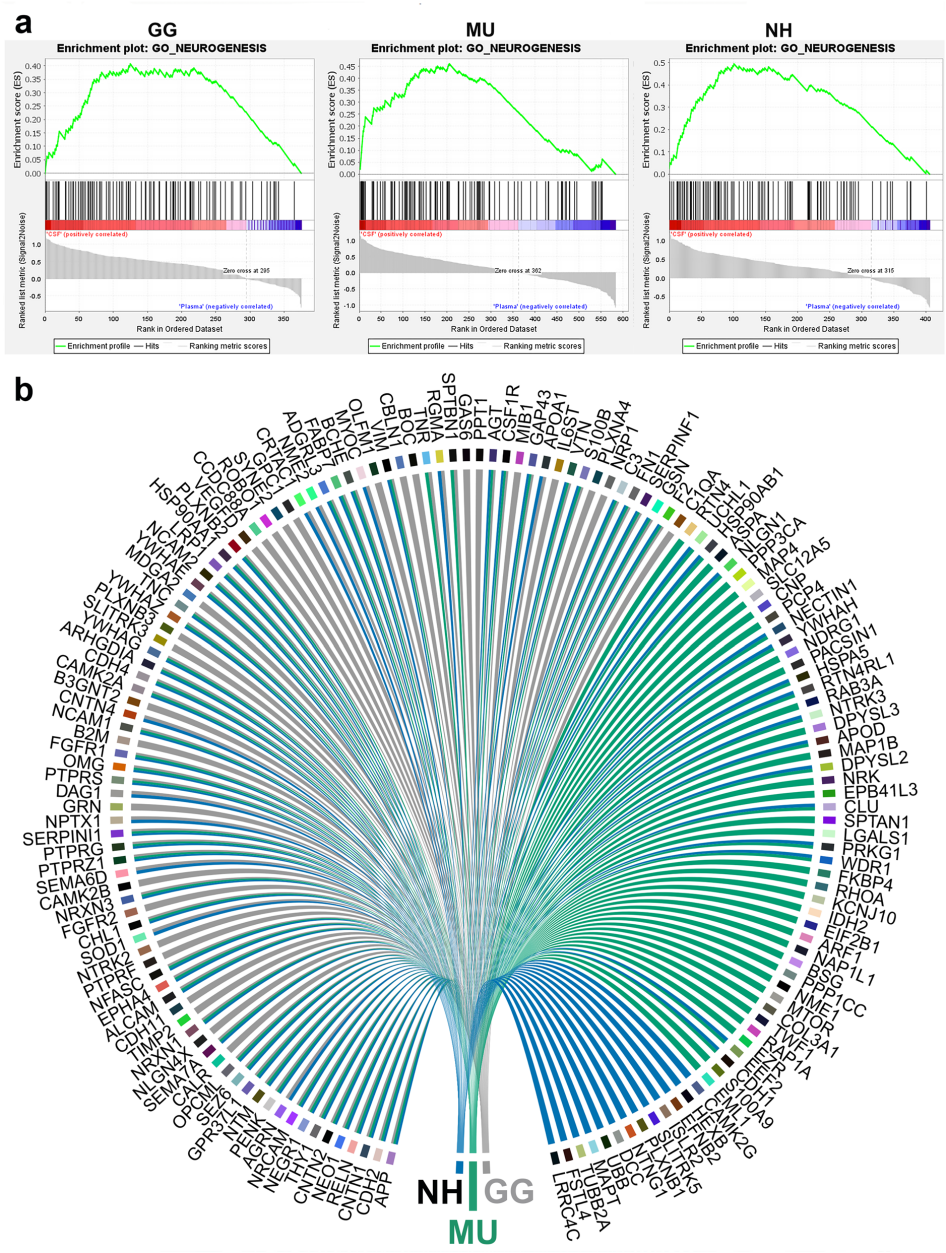
Gene set enrichment analysis (GSEA) and comparison of cerebrospinal fluid and plasma proteomes reveals neurogenesis in all studied avian species. (**a**) GSEA plots of the GO term Neurogenesis for chicken (GG), budgerigar (MU) and cockatiel (NH) in cerebrospinal fluid compared to plasma (GSEA version 4.1.0, http://www.gsea-msigdb.org) (**b**) Circular plot representation of proteins identified by GSEA with the GO term Neurogenesis for GG, MU and NH. The proteins are labelled by their gene codes (R version 4.0.0, www.r-project.org).

This is consistent with the result of the gene set enrichment analysis (GSEA) based on GO Biological functions classification and pathway enrichment in CSF compared to plasma that revealed differential representation in proteins involved in the neural function. Enriched pathways in plasma were mostly related to immune functions. Supplementary Table S5 shows the complete list of the significantly enriched pathways at nominal *p* value < 0.05 and FDR < 0.25 in CSF and PL, and Supplementary Figs. S7–S10 the graphical representation of their gene overlap. The GSEA analysis revealed several gene sets linked to neurogenesis, neural development and differentiation which were preferentially expressed into CSF and shared across the species (Fig. 7). Figure 8 shows the nominal enrichment scores for GO term Neurogenesis and the genes implicated for each species.

## Discussion

CSF connects the bloodstream with the CNS and reflects the physiology of the brain. Here we have described the adult CSF and PL protein composition in three avian species (chicken, budgerigar, and cockatiel) using gel-free nLC-MS/MS. First, we have confirmed that the phylogenetically closest avian reference proteome yields the highest efficacy in protein identification success rates, although a proportion of proteins (7.2%, 18% and 13.8% for GG, MU and NH, respectively) was mapped only using other references. In total, following the data filtering, we identified in the CSF and PL 483 proteins in the chicken, 641 proteins in the budgerigar and 458 proteins in the cockatiel. GO pathway analyses of the core proteomes revealed components of several signalling and metabolic processes such as regulation of peptidases, synapse organisation, regulation of IGF transport and uptake by IGFBPs in CSF and blood coagulation, complement activation and glycolysis in PL. Comparative analysis of the proteomes derived from the two biological fluids showed that the CSF and PL are clearly distinguishable in terms of their protein content. In CSF, the pathways involved in neurogenesis, and neural development and differentiation are over-represented when compared to blood plasma.

Current research increasingly adopts investigation in diversified taxa lacking complete and annotated genomes and proteomes. In this study, we investigated also the cockatiel, a species currently lacking a genome sequence. We found that by cross-species protein identification using the sequence database from its evolutionarily closest relative, the budgerigar, we could identify 458 proteins represented in the CSF and PL. This is more than if other reference genomes were used (chicken, 327; zebra finch, 291). This trend was consistent with our findings in other species included in this study. Interestingly, however, some proteins were identified only when mapped against the other mapping combinations. Quantitative proteomics of unsequenced organisms is still a challenge and, when de novo sequencing of the unidentified proteins is unavailable, the reference proteome of a close-related phylogenetically species is utilised to identify the proteome in question. An analogous approach has been used to characterise the proteome of unsequenced micro-organisms and *Xenopus laevis* microtubule-associated proteome^39, 40^, but this is the first study reporting a paradigm of cross-species quantitative proteomics in diverse avian taxa.

We found more proteins in CSF compared to plasma, a finding which was consistently found in all avian species in this study and similar to what has been previously reported for humans in paired samples^41, 42^. In a cohort of more than 100 individuals, Dayon et al. identified 790 and 422 proteins in parallel CSF and PL samples respectively with an overlap of 255 proteins shared by both fluids^41^. In a previous study, a maximal protein set of 3,081 and 1,050 CSF and PL proteins was identified respectively with 877 proteins common in both fluids from a group of 21 healthy individuals^42^. The reasons for the differences in the number of protein identifications between these two studies could be attributed in different sample preparation approaches and detection techniques. Thus, although the protein concentration is likely higher in PL compared to CSF^43^, the variety of proteins is greater in the CSF compared to PL, being enriched from ca. 20% with proteins expressed in the choroid plexus and brain^44^. Blood and serum comprise receptor ligands, immunoglobulins, tissue leakage products, aberrant secretions and proteins that are extraneous to the animal. Many blood proteins are synthesized in liver^45^. Although quality of CSF collection can influence the protein content observed, the CSF concentration of CNS-derived proteins remains unaffected following contamination of the fluid with blood^46^. In the present dataset, we found the presence of haemoglobin subunits and carbonic anhydrase that can be attributed to some minor blood contamination^47^. Yet, like in Smith et al.^13^, although haemoglobin subunits were found amongst the most CSF abundant proteins, our clustering analysis clearly differentiated CSF and plasma samples in all studied individuals and 58.5% of the shared proteins across species, such as gamma-enolase, neuronal growth regulator 1 or neural cell adhesion molecule 1, could be identified selectively in CSF.

Using gel-based techniques and mass spectrometry, the proteomes of eCSF from rat, chicken and human have been previously described to elucidate the molecular composition of CSF and its biological role during embryonic neurogenesis^10–12, 48^. Human eCSF contained 188 proteins, 44% of those also present in rat eCSF. Functional analysis revealed protease inhibitors, extracellular matrix proteins, and transport proteins as well as signalling and intracellular proteins^12^. Comparing the data available for human eCSF with our results in birds, 39%, 50% and 47% of the proteins overlap with adult CSF from chicken, budgerigar and cockatiel, respectively. In chicken embryos investigated at the developmental stage HH24, using 2D-PAGE, 26 proteins were identified, related to the extracellular matrix, regulation of the osmotic pressure and metal transport, cell survival, transport of retinol and vitamin D as well as mitogen-activated protein kinase activators, antioxidant and antimicrobial proteins, intracellular proteins and some unknown proteins. We found that 50% of these proteins were included also in our data. Many of these proteins regulate the development in systems other than CNS, while others are altered during neurodegeneration^10, 11^.

LC-based methods allowed the identification of a dramatically increased number of proteins in CSF. Most recently, using LC–MS/MS after abundant protein depletion, Macron et al. identified 3,174 proteins in a commercial pool of “normal” human CSF samples^15^. However, expectedly, interindividual variation is accountable for identification of many of the rare proteins. For example, out of 2,615 CSF proteins, only 20% of the identified proteins were shared by all six healthy subjects^16^. In a combined dataset from three studies the full human CSF proteome of 4814 proteins was revealed, but only about 40% of these proteins were common. We found that about a half of the proteins identified in either plasma or CSF in each species showed a high degree of inter-individual variability, while the other half was relatively consistently represented across all individuals. In our study we have shown that 71.22 and 55.27% (GG), 41.97 and 45.31% (MU), and 56.92 and 38.87% (NH) proteins identified for CSF and PL respectively were common to all individuals within species and only 193 proteins were common across all three species. Thus, the number of proteins that are individually specific is high. The reasons for this variation, however, remain unclear^42, 49, 50^. The molecular variations in CSF composition can be possibly increased by post-translational modifications, age, blood contamination^47^ and circadian rhythm^51^.

We also found proteins of intracellular origin in the CSF, unlike in PL. These were consistently observed in human, rat and chick CSF/eCSF and classified mainly as “binding proteins”^10, 12, 52–54^. It has been suggested that these proteins are waste products eliminated from the brain extracellular space by the CSF. A small proportion could also indicate cellular contamination of the samples^54^. Post-mortem samples have been previously reported to contain also cytoskeletal proteins, glycolysis and antioxidant enzymes released into the CSF following cell necrosis^55^.

Our comparative analysis shows that CSF and plasma were distinguishable based on their proteome compositions. From the proteins over-represented in CSF compared to plasma, such as homologs of neuronal growth regulator 1 (*NEGR1*) and reelin (*RELN*), 72 were shared across all the three avian species. A PPI network constructed based on these proteins, as well as the enrichment analysis of biological functions revealed namely representation of pathways associated with cell adhesion, nervous system development and neurogenesis. In addition, using GSEA, we indicated in CSF enrichment of pathways involved in neural functions across the studied species. In addition to those revealed by differential analysis, the CSF samples shared several gene products related to neurogenesis for example neogenin 1 (*NEO1*), neurofascin (*NFASC*), neuromodulin (*GAP43*), or pigment epithelium-derived factor (*SERPINF1*). Our results suggest that adult neurogenesis occurs in all the avian species studied. Previous reports have stressed the importance of various growth factors present in CSF which bind to receptors located on the apical membrane of cortical progenitor cells and promote the development and growth of neural stem cells and cortical explants^9, 56^. In this study, we identified proteins such as 14–3-3 proteins (*YWHAG , YWHAZ*) and fibroblast growth factor receptor 1 (*FGFR1-4*) participating in the fibroblast growth factor pathway^57^, molecules interacting with the Wtn^9^ pathway such as the Wtn inhibitor Dickkopf-related protein 3 (*DKK3*) and cadherins (*CDH13*, *CDH2*, *CDH6, CDH4*), amyloid beta A4 protein precursor (*APP*)^12^, insulin-like growth factor II (*IGF2*) and insulin-like growth factor-binding proteins (*IGFALS*, *IGFBP2*, *IGFBP7*)^9, 58^. However, how these proteins are regulated in the context of systems level, their lifetime in CSF, as well as the contribution of interindividual variability requires further investigation.

In vitro studies of adult human CSF treatment in neural stem cells showed that adult human CSF promoted gliogenesis, but not neurogenesis^59, 60^. Despite ontogenetic differences in CSF protein composition in its ability to induce neurogenesis, the neurogenic potential is preserved in the adult mammalian brain and can be activated only by external stimuli including exercise, signalling molecules following injury or eCSF^6, 61^. In contrast, in birds, neuronal cell proliferation occurs in the subventricular zone of the lateral ventricle, and migration to widespread regions of telencephalon follows^62^. Immunohistochemical studies have demonstrated adult neurogenesis in the telencephalon and other brain regions of canaries^63^, marsh tits^64^, ring doves^65^, chicken^66^, pigeons^67, 68^, parrots^69^, ostrich and emu^70^. While interspecific differences in adult neurogenesis likely exist, it is well established that factors such as age, environmental complexity, seasonal variation, hormones, stress, rearing conditions, physical activity, social isolation and social complexity influence its outcome^18^. For example, the protein expression of various neurotrophic factors such as nerve growth factor, insulin-like growth factor 1, vascular endothelial growth factor, and brain-derived neurotrophic factor may alter upon increased physical activity or dietary restriction^71^. It is difficult to analyse the effects of the above-mentioned factors across species in this study, because the species examined have different developmental modes (precocial and nidifugous chickens vs. altricial and nidicolous parrots). The functional role of adult neurogenesis in birds remains unknown. It has been proposed to be associated with continuous learning. In adult mammals, the proliferative hot spots may disappear altogether^62^, but comparative approaches between birds and mammals have elucidated similarities on the origin of neurons, the phenotype and proliferative mechanisms of stem cells and the migration and differentiation of neurons as well as differences such as the spatial distribution of adult-born neurons, their mode of migration and phenotypic diversity^3, 69, 72^. We found that chicken and parrots share common proteins and pathways associated with the modulation of neuronal cell proliferation and plasticity in CSF. Being based on a single methodological approach, our identification of these gene sets and pathways in birds serves for an initial insight and subsequent experimental verification is required. Nevertheless, our study forms an essential reference for further molecular analysis.

## Conclusion

The advent of the proteomic techniques has allowed new insights into molecular processes driving the CNS function, with research in CSF forming its cornerstone. For the first time, we concurrently explored in birds the proteomes of two main biological fluids, PL and CSF. Importantly, we focused on multiple species, the chicken and two parrot species, the budgerigar and cockatiel, investigating adult individuals. The proteins over-represented in CSF were mainly classified into functional pathways involved in neural function, providing a novel molecular evidence for adult neurogenesis in birds. Consistently with histological evidence for adult neurogenesis in birds, understanding the molecular components involved in this process is essential for designing future experimental work in this system.

## Methods

### Sample collection

CSF and PL samples were collected from a total of seven chicken, five budgerigars and five cockatiels. The animals were originated from legal imports for the pet trade (Prague, Czech Republic) who confirmed their status as healthy, adult birds and were kept in our animal facility (accreditation No. 37428/2019-MZE-18134). The animals were euthanised by CO_2_ and mounted on a stereotaxic instrument (Stoelting Co., Illinois, USA). After incision in the dorsal neck region and exposure of the skull occipital region, a needle of a 0.5 ml sterile insulin syringe was inserted into cisterna magna^73^. Approximately 3–10 µl of clear, colourless CSF was collected per animal and stored at −80 °C until analysis preparation. Blood was extracted from the carotid artery and PL obtained by centrifugation (14,000 g) was stored at −80 °C, until further use. Experimental design involving animals was performed by accredited researchers (EV: CZ03814, MV: CZ02568). The research was approved by the Ethical Committee of Charles University, Faculty of Science (permits MSMT-1373/2016-4 and MSMT-30397/2019-5) and was carried out in accordance with the current laws of the Czech Republic and European Union and in compliance with the ARRIVE guidelines.

### Protein extraction and precipitation

The ProteoSpin detergent-free total protein isolation kit (Norgen Biotek, Thorold, ON, Canada) was used for isolation and purification of the total protein content from CSF and PL according to the manufacturer's instructions and then frozen at −20 °C. Protein precipitation was performed using acetone (1/5, acetone/protein) at −20 °C for 1 h. All precipitated samples were centrifuged at 14,000 g and 4 °C for 15 min. After centrifugation, the supernatant was discarded, the samples dried for 30 min at 37 °C and were cleaved with trypsin (i.e., 1/50, trypsin/protein) at 37 °C overnight before mass spectrometry analysis.

### nLC − MS/MS analysis

Nano reversed-phase columns were used to elute peptide cations using a previously described method^74^. The eluting peptide cations were converted to gas-phase ions by electrospray ionization and analysed on a Thermo Orbitrap Fusion mass spectrometer (Q-OT-qIT, Thermo). Survey scans of peptide precursors from 350 to 1400 m/z were performed at 120 K resolution (at 200 m/z) with a 5 × 105 ion count target. Tandem MS/MS was performed by isolation at 1.5 Th with the quadrupole, high-energy collision dissociation fragmentation with a normalized collision energy of 30 and rapid scan MS analysis in the ion trap. The MS/MS ion count target was set to 104 and the max injection time was 35 ms. Only those precursors with a charge state of 2–6 were sampled for MS/MS. The dynamic exclusion duration was set to 45 s with a 10-ppm tolerance around the selected pre-cursor and its isotopes. Monoisotopic precursor selection was turned on and the instrument was run at top speed with 2 s cycles.

### Proteome data analysis

All data were collected and quantified using MaxQuant software version 1.6.10.43^75^. False discovery rate (FDR) was set to 1% for identification of all peptides and proteins. We set a minimum peptide length of seven amino acids. The Andromeda search engine was used for the MS/MS spectra search against the chicken *Gallus gallus* UniProt reference proteome (downloaded on March 2020, containing 27,540 entries), budgerigar *Melopsittacus undulatus* UniProt reference proteome (downloaded on February 2020, containing 23,704 entries) and zebra finch *Taeniopygia guttata* UniProt reference proteome (downloaded on February 2017, containing 23,455 entries), with all duplicates removed. Enzyme specificity was set as C-terminal to Arg and Lys, also allowing cleavage at proline bonds^76^ and a maximum of two missed cleavages. Dithiomethylation of cysteine was selected as a fixed modification and N-terminal protein acetylation and methionine oxidation as variable modifications. Quantifications were performed with the label-free quantification algorithms using a combination of unique and razor peptides^75^. The mass spectrometry proteomics data have been deposited to the ProteomeXchange Consortium via the PRIDE^77^ partner repository with the dataset identifier PXD021633.

### Mapping approaches

Annotated reference proteomes are presently available for the chicken^78^ and the budgerigar^79^, but not for the cockatiel. Thus, mapping cockatiel reads on its own reference proteome was not possible. To estimate the effect of this condition, we compared the MS/MS identification success rates of chicken, budgerigar and cockatiel proteomes, each mapped against three applicable avian reference proteomes, the chicken, budgerigar and zebra finch. We performed the following data filtering to eliminate low-scoring spectra: from further analysis we excluded all the unlabelled peptides, peptides only identified by site or reverse, all contaminants, proteins identified by < 2 peptides and all proteins occurring in less than 1/3 of the samples for each group. For the purpose of comparison, chicken ortholog gene codes were assigned to all identified proteins using the database OrthoDB (v10.1)^80^. When the gene code was not possible to retrieve, the UniProt protein ID was used.

For protein classification, we used the online Protein Analysis Through Evolutionary Relationships (PANTHER) library^81^. To find overrepresented Gene Ontologies (GOs)^82^ in the core proteomes of CSF and PL, we launched the g:profiler against the chicken reference list (all genes in the database) and the annotation data sets of the Gene Ontology and biological pathways (KEGG, REACTOME)^83–85^. The Benjamini–Hochberg FDR threshold was set to 0.05 of all identified proteins in PL and CSF of chicken, budgerigar and cockatiel.

### Statistical analysis

The statistical and bioinformatic analysis conducted in the R software^86^, Microsoft Excel and Perseus software platform (http://www.perseus-framework.org; Max Planck Institute of Biochemistry, Martinsried, Germany). The Venn diagrams were prepared using Biovenn^87^ and Jvenn^88^. In order to remove minor technical variation between samples, the abundances of the identified proteins were previously normalised using the Variance stabilization normalization method^89, 90^ and DEP 1.12.0 R package. Missing data were imputed using random draws from a manually defined left-shifted Gaussian distribution applying the default settings of Perseus software. PCA was performed to examine the variation between the PL and CSF fluids in these species using Perseus and R. Significantly differentially represented proteins were identified by a Student’s t-test corrected by the Permutation Based FDR (FDR-adjusted *p* value < 0.05). In order to visualise biological protein groups clustered together in either CSF or PL, hierarchical cluster analysis was performed in Perseus using default parameters (Euclidean distance with average linkage, 300 clusters, 10 iterations, 1 restart and k-means pre-processing for both row and column tree). To indicate the proteins significantly differentially represented in CSF and PL in the three studied species, a Volcano Plot analysis was performed in Perseus (unpaired Student’s T-test, S0 = 2, permutation-based FDR) and R. In the volcano plot, protein intensity fold-change between the fluids is represented as log_2_. The Retrieval of Interacting Genes (STRING 11.0; http://string-db.org)^91^ web-tool was used to analyse and construct the PPI network of the common group of proteins across the three species that were over-represented in CSF compared to PL (adjusted *p* value cut off (FDR) < 0.05, identified by the t-test analysis described above) . The interaction sources included experimental data and significant PPIs were considered those with a combined score > 0.4 and annotations from human (*Homo sapiens*). For the network visualization, also using annotations from *Homo sapiens*, we used ShinyGO (v0.61)^92^ online tool to determine the GO terms of biological functions which are significantly overrepresented in CSF compared to blood PL in the same set of common gene products over-represented in the CSF compared to PL. GSEA was also performed. All proteins were ranked based on the association between their abundance and the class distinction (CSF or PL), using annotations from *Homo sapiens* (Human Gene Symbol with Remapping MSigDB.v7.2). We used the GOs analysis of biological processes, nominal *p* value < 0.05 and adjusted *p* value < 0.25, as previously suggested. An FDR of < 25% is recommended for discovering candidate gene sets to be further validated as a result of future research^93^.

## Supplementary Information

Supplementary Information

Supplementary Table S1. nLC-MS/MS output from different mapping approaches.

Supplementary Table S2. Core proteomes of CSF and PL.

Supplementary Table S3. GO Classifications and Pathway analyses of CSF and PL.

Supplementary Table S4. Differentially represented proteins in CSF vs plasma.

Supplementary Table S5. GSEA results.

## Data Availability

The mass spectrometry proteomics data have been deposited to the ProteomeXchange Consortium via the PRIDE^77^ partner repository with the dataset identifier PXD021633.
