# Supplementary material for: Comparison of plasma and cerebrospinal fluid proteomes identifies gene products guiding adult neurogenesis and neural differentiation in birds
Source: Sci Rep. 2021 Mar 5;11:5312. doi: 10.1038/s41598-021-84274-x (PMC7935914; doi:10.1038/s41598-021-84274-x)
Supplement: Supplementary file 1 — Supplementary Information [file 41598_2021_84274_MOESM1_ESM.pdf]

Supplementary materials

Comparison of plasma and cerebrospinal fluid proteomes identifies gene products guiding adult neurogenesis and neural differentiation in birds

Authors: Eleni Voukali[1*], Nithya Kuttiyarthu Veetil[1], Pavel Němec[1], Pavel Stopka[1] and Michal Vinkler[1]

Addresses:

1) Charles University, Faculty of Science, Department of Zoology, Viničná 7, 128 44 Prague, Czech Republic, EU

*Corresponding authors: Eleni Voukali, e-mail: voukalie@natur.cuni.cz, evoukali@yahoo.gr, tel.: +420221951845, Michal Vinkler, e-mail: michal.vinkler@natur.cuni.cz, tel.: +420221951845.

Correspondence address:

Charles University, Faculty of Science, Department of Zoology, Viničná 7, 128 44, Prague, Czech Republic, EU

Competing interests

The authors declare no competing interests.

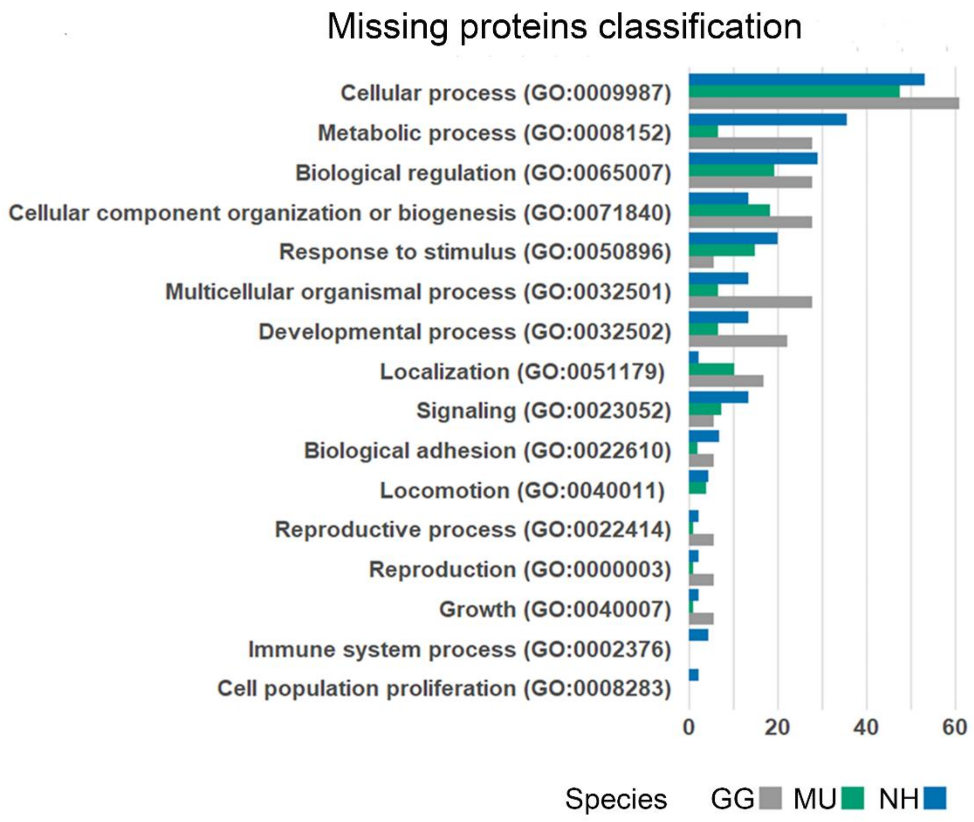

Figure S1|Bar graph showing the gene ontology (GO) classification for biological function based on the *Gallus gallus* genome annotation for proteins that were not identified when the CSF and PL spectra were mapped against the proteome of the same species (GG x GG and MU x MU) or a related (in the case of NH x MU) proteome, but were identified when mapped against the other avian reference proteomes. GG identities are shown in grey, MU in green and NH in blue; the length of each bar shows the relative number of genes in each protein subset against the total number of missing proteins for each species (R version 4.0.0, www.r-project.org).

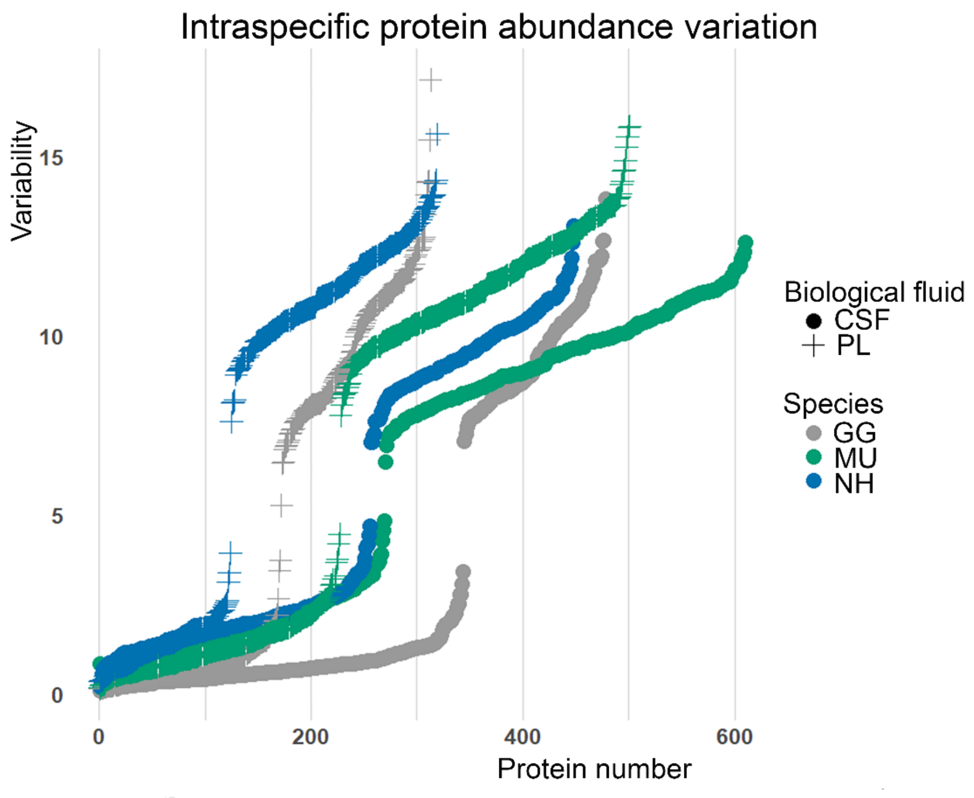

Figure S2| Summary variation of the proteomes from cerebrospinal fluid (CSF) and plasma (PL) in chicken (GG) and two parrot species, the budgerigar (MU) and cockatiel (NH). The intraspecific protein variability of abundance measurements in CSF (filled circles) and PL (crosses) samples is shown for GG (grey, n=7), MU (green, n=5) and NH (blue, n=5). Proteins were ordered by their increasing standard deviation (R version 4.0.0, www.r-project.org).

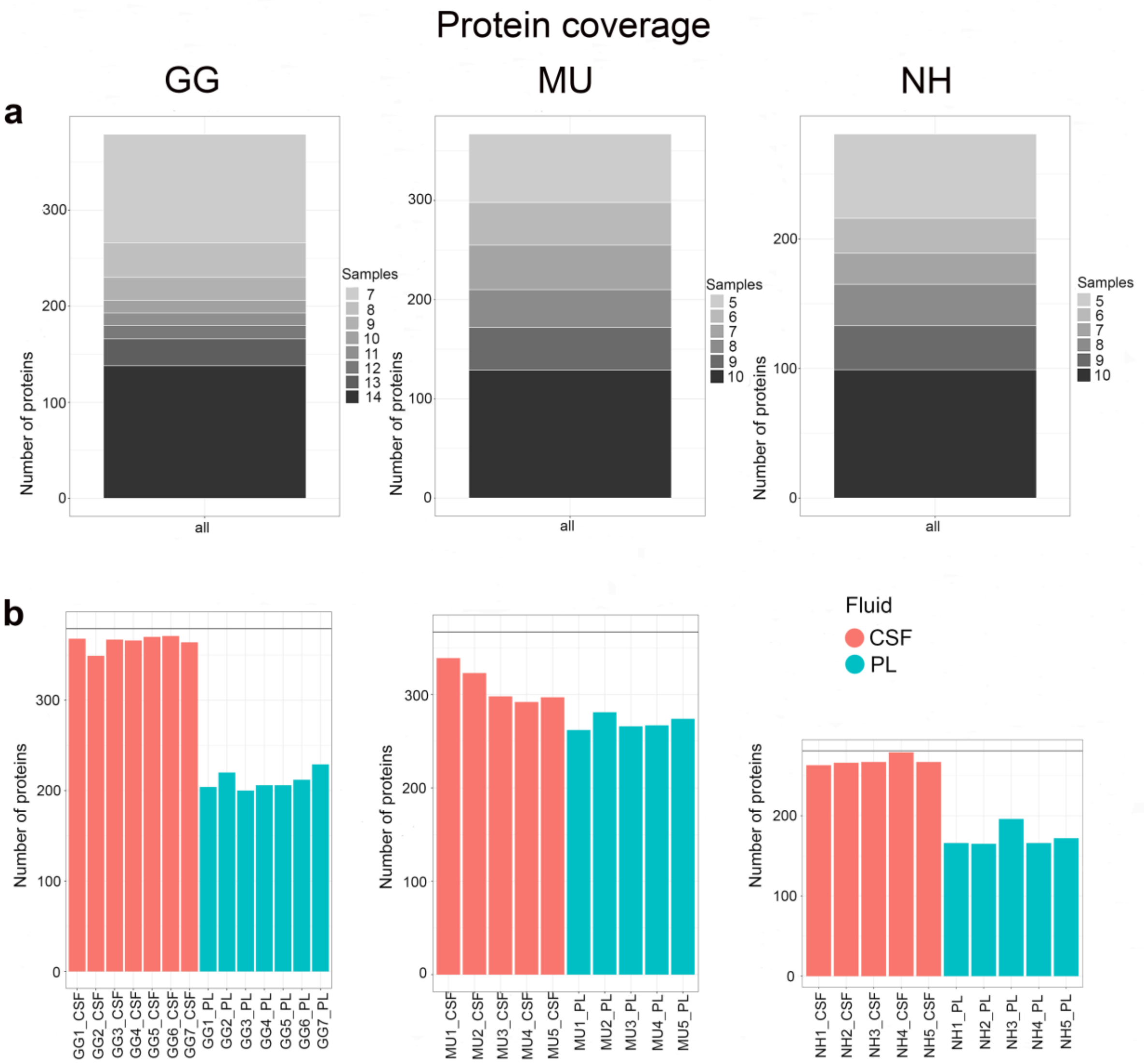

Figure S3| Protein coverage of the full cerebrospinal fluid (CSF) and plasma (PL) proteomes. **(a)** The overlap of the protein identifications is plotted for both CSF and PL for chicken (GG, n=14), budgerigar (MU, n=10) and cockatiel (NH, n=10). The x-axis shows the stratification based on the number of samples and the y-axis indicates the number of proteins covered for each stratum. **(b)** The number of identified proteins per sample is illustrated in the barplots for GG (left-hand side), MU (middle) and NH (right-hand side). The length of each bar shows the number of proteins in each sample (R version 4.0.0, www.r-project.org).

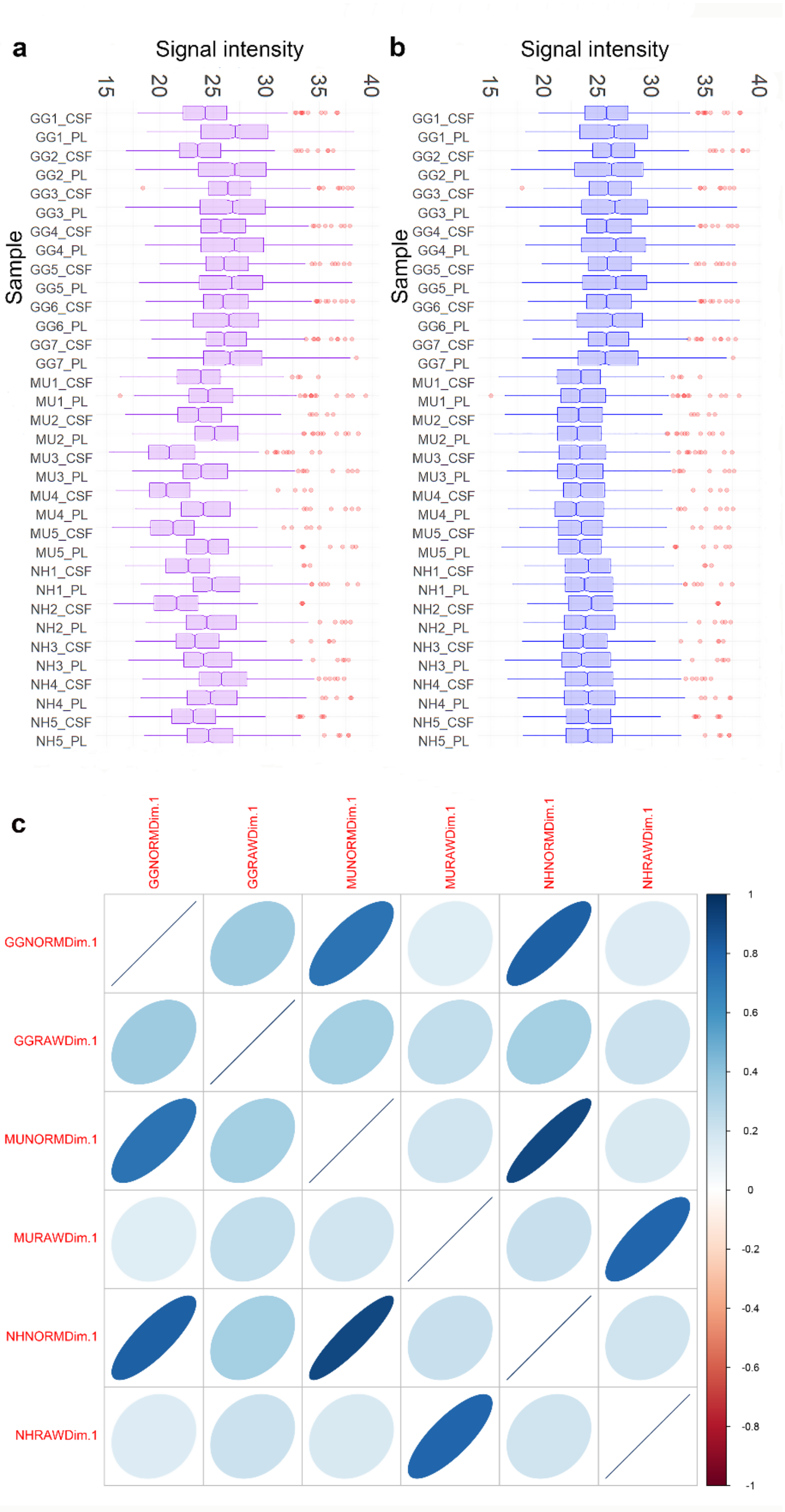

Figure S4| Inspection of raw and normalised data. **(a)** The distributions of log2 signal intensities indicate that the data revealed some variation between individuals before normalization. However, after the variance stabilization normalization **(b)**, the boxplots show almost no variation between the samples. **(c)** Correlation matrix of the principal component 1 (Dim.1) for each dataset. The bar on the right scales the positive correlations displayed in blue and negative correlations in red colour, indicating that raw and normalised data are only positively correlated. Colour intensity and the size of the circle are proportional to the correlation coefficients. GGRAW (chicken raw data), GGNORM (chicken normalised data), MURAW (budgerigar raw data), MUNORM (budgerigar normalised data), NHRAW (budgerigar raw data), NHNORM (cockatiel normalised data) (R version 4.0.0, www.r-project.org).

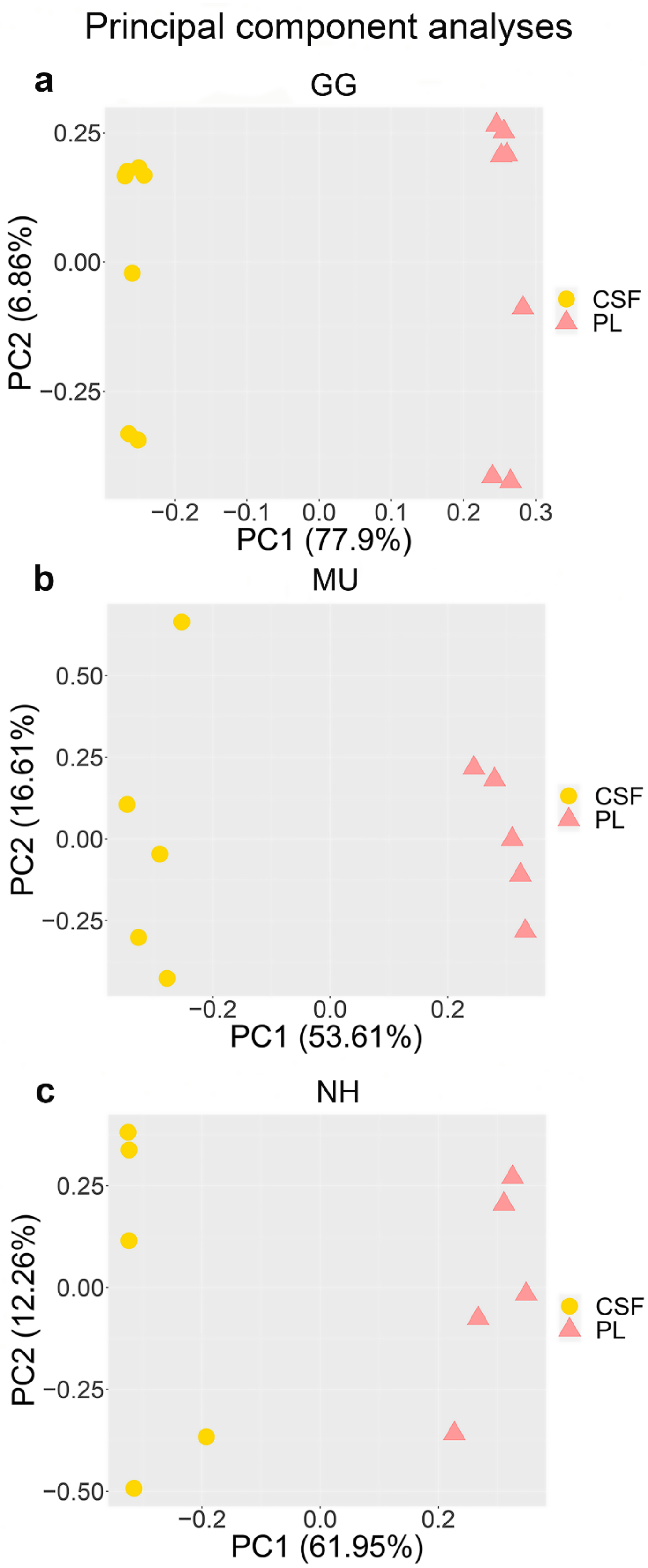

Figure S5| Principal component analyses (PCA) showing preferential grouping between cerebrospinal fluid (CSF) and plasma (PL). PCA shows clusters differentiating between CSF (yellow dots) and PL (red triangles) for **(a)** chicken (GG; Component 1, 77.96%), **(b)** budgerigar (MU, Component 1, 51.65%), and **(c)** cockatiel (NH, Component 1, 46.84%) and shows that the proteomes of the two fluids were distinguished in all studied species (R version 4.0.0, www.r-project.org).

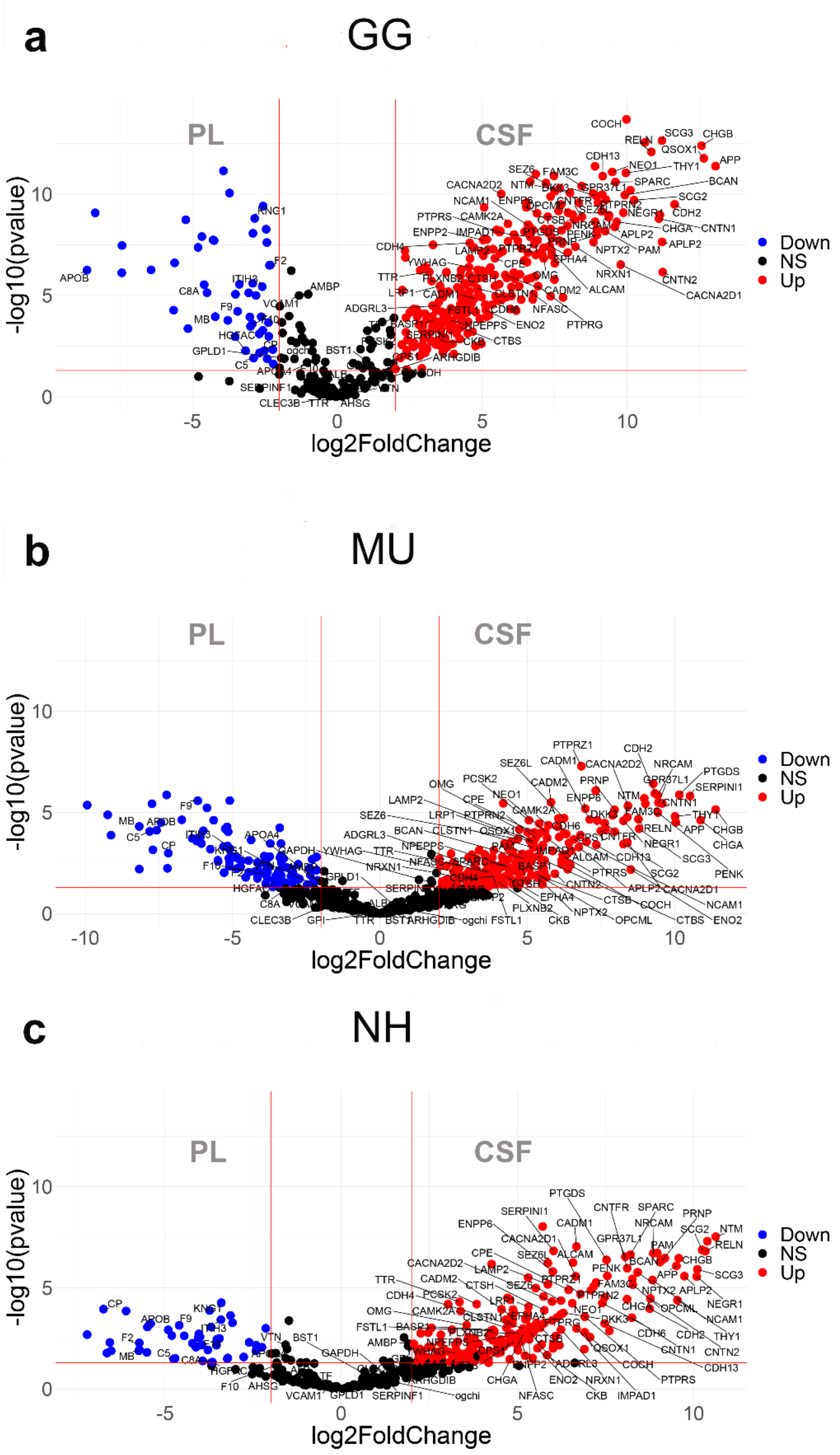

Figure S6| Volcano plots of the fold changes on p-values between all identified proteins in cerebrospinal fluid (CSF) and plasma (PL) using t-test. The fold differences of proteins (x-axis) dependent on their p-values (y-axis) are shown in dots colour-coded in blue, red and black, demonstrating the significantly under-represented proteins (Down), over-represented proteins (Up), and non-significantly represented proteins (NS, $p<0.05$, fold change cut -off ≥ 2) respectively in CSF compared to PL for **(a)** chicken (GG), **(b)** budgerigar (MU) and **(c)** cockatiel (NH). The commonly differentiated proteins across species are indicated using their gene codes (R version 4.0.0, www.r-project.org).

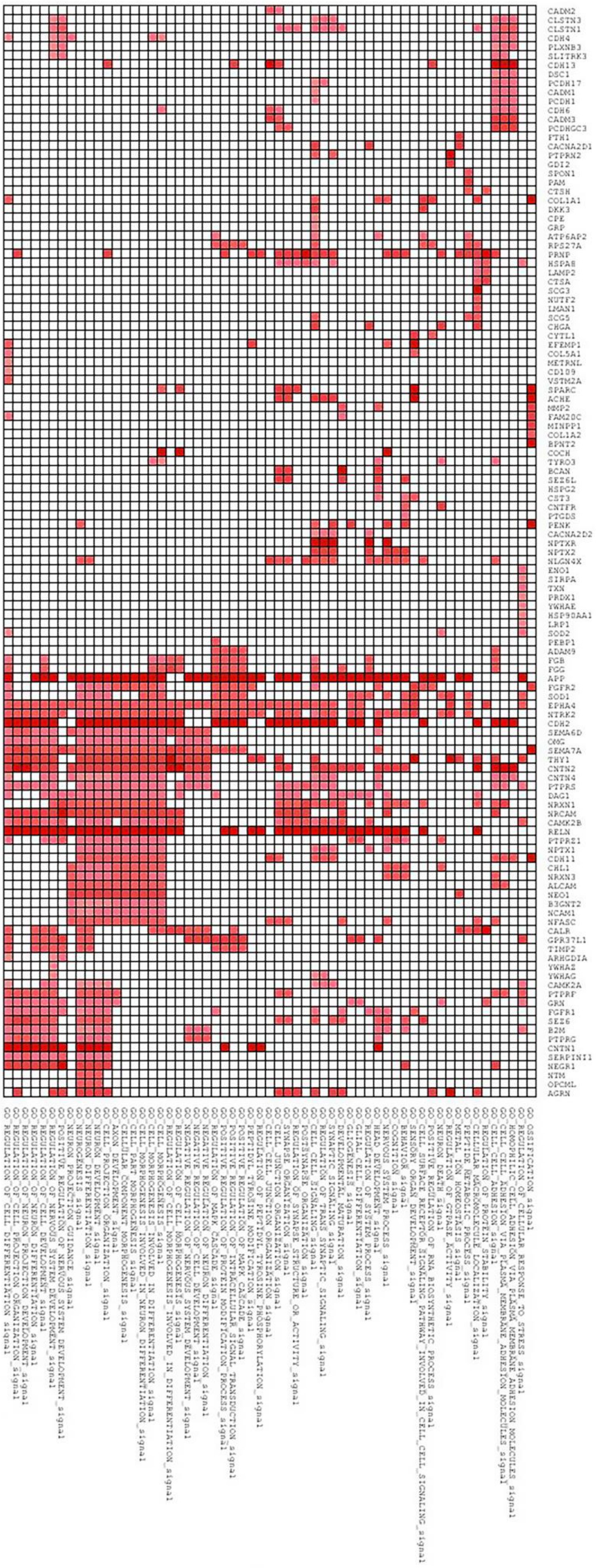

Figure S7| Leading edge analysis showing the overlap between the significant gene sets in cerebrospinal fluid of chicken. The gene sets/pathways with nominal p-value<0.05 and FDR q-value<0.25 are listed on the bottom and gene codes on the right correspond to the subset of genes that contributes most to the enrichment result. The intensity of the red colour indicates the degree of the positive nominal enrichment score (GSEA version 4.1.0, http://www.gsea-msigdb.org).

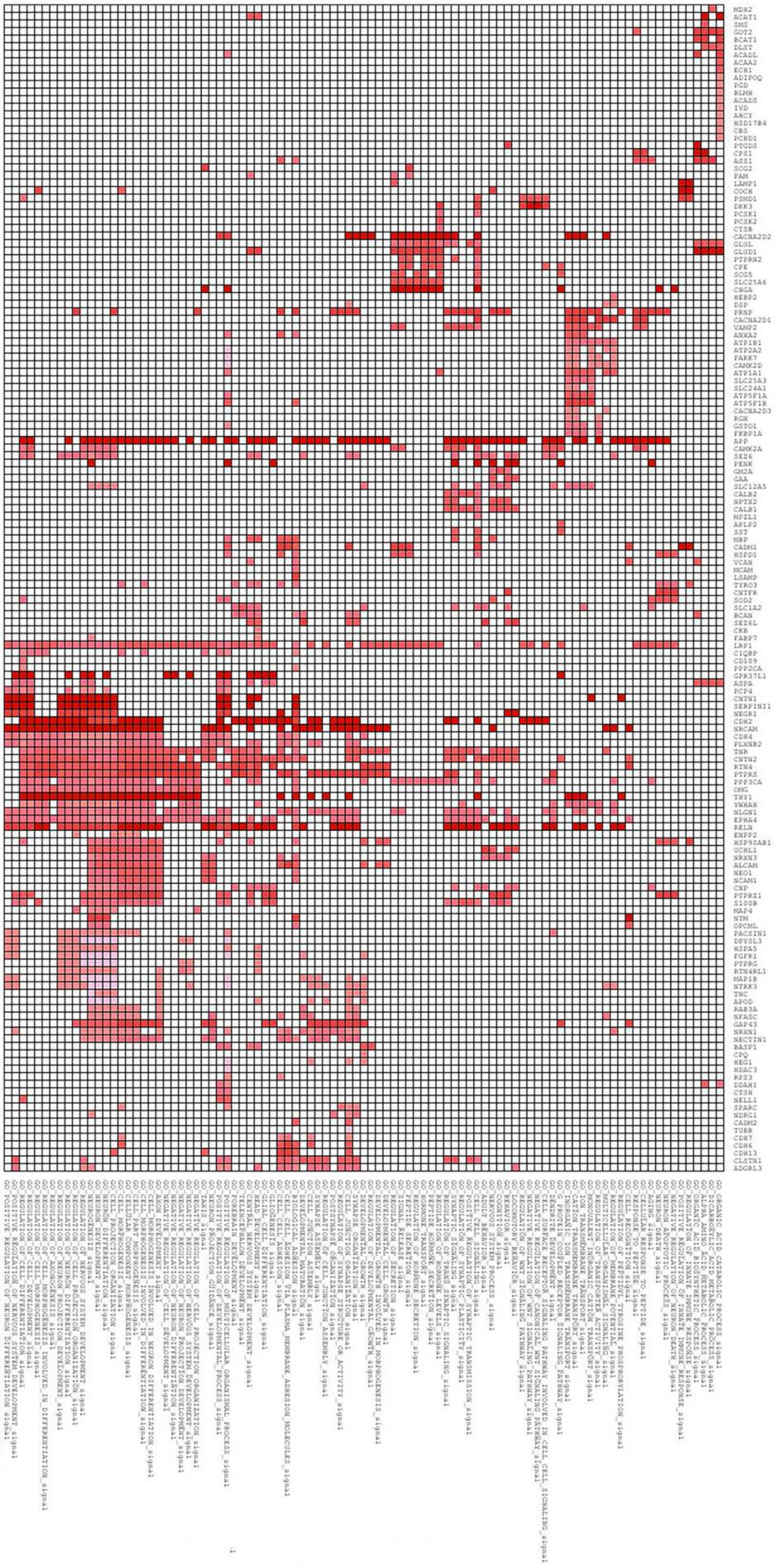

Figure S8| Leading edge analysis showing the overlap between the significant gene sets in cerebrospinal fluid of budgerigar. The gene sets/pathways with nominal p-value<0.05 and FDR q-value<0.25 are listed on the bottom and gene codes on the right correspond to the subset of genes that contributes most to the enrichment result. The intensity of the red colour indicates the degree of the positive nominal enrichment score (GSEA version 4.1.0, http://www.gsea-msigdb.org).

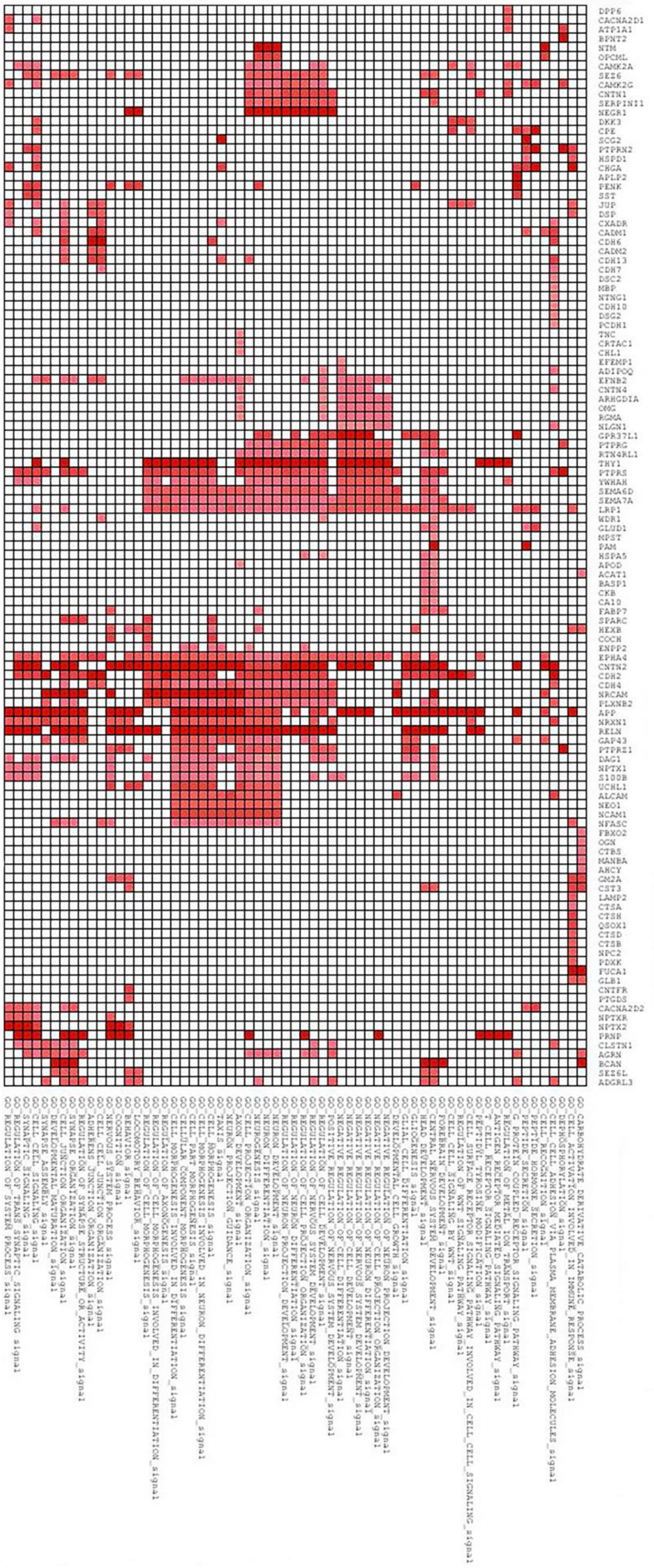

Figure S9| Leading edge analysis showing the overlap between the significant gene sets in cerebrospinal fluid of cockatiel. The gene sets/pathways with nominal p-value<0.05 and FDR q-value<0.25 are listed on the right and gene codes upfront correspond to the subset of genes that contributes most to the enrichment result. The intensity of the red colour indicates the degree of the positive nominal enrichment score (GSEA version 4.1.0, http://www.gsea-msigdb.org).

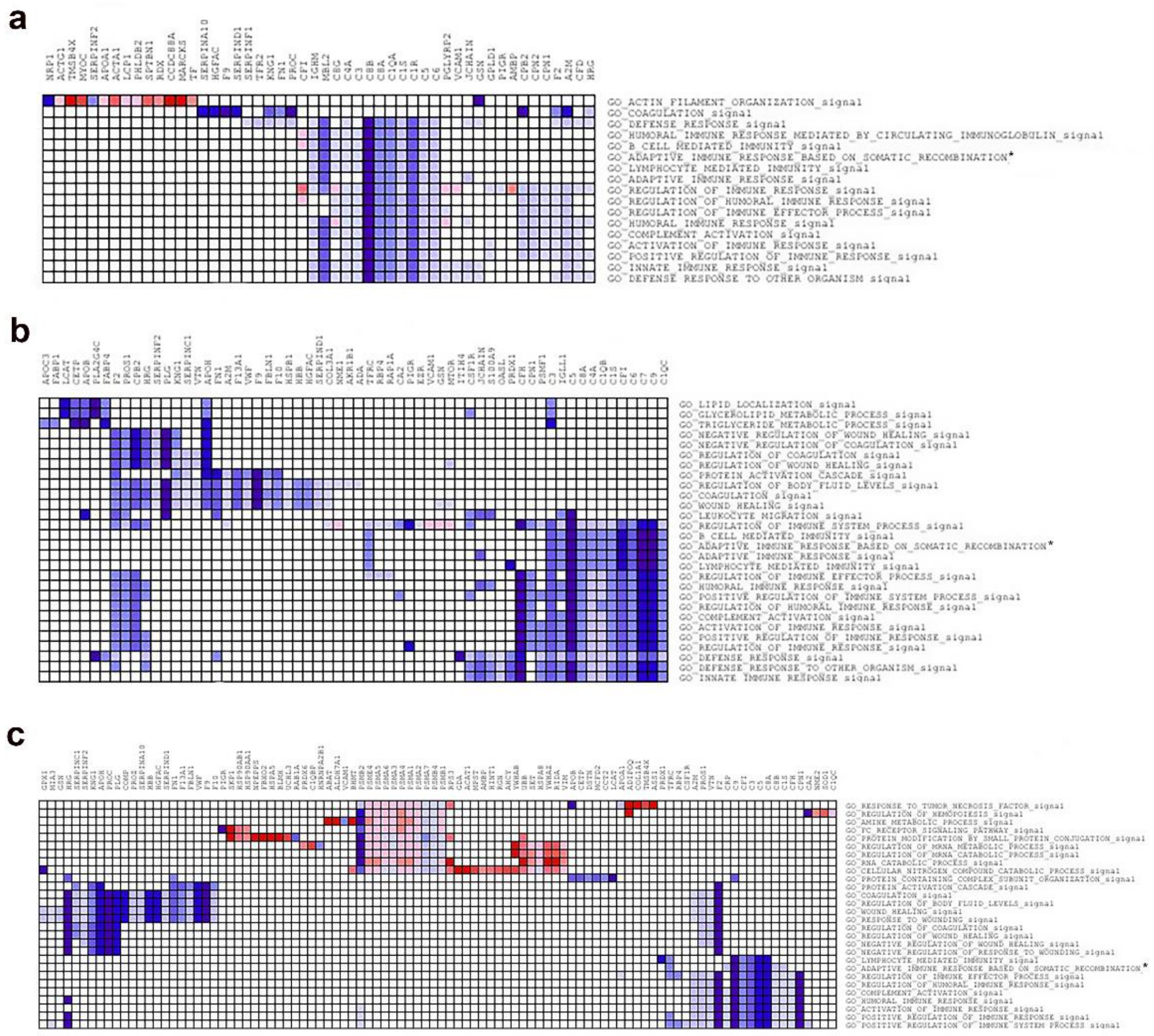

Figure S10| Leading edge analysis showing the overlap between the significant gene sets in plasma. The gene sets/pathways with nominal p-value<0.05 and FDR q-value<0.25 are listed on the right and gene codes upfront correspond to the subset of genes that contributes most to the enrichment result for **(a)** chicken, **(b)** budgerigar and **(c)** cockatiel. The intensity of the blue colour indicates the degree of the negative nominal enrichment score and that of red colour the positive nominal enrichment score (GSEA version 4.1.0, http://www.gsea-msigdb.org). *GO_ADAPTIVE_IMMUNE_RESPONSE_BASED_ON_SOMATIC_RECOMBINATION_OF_IMMUNE_RECEPTORS_BUILT_FROM_IMMUNOGLOBULIN_SUPERFAMILY_DOMAINS.
